# Evaluation of the Use of Antibiofilmogram Technology in the Clinical Evolution of Foot Ulcers Infected by *Staphylococcus aureus* in Persons Living with Diabetes: A Pilot Study

**DOI:** 10.3390/jcm10245928

**Published:** 2021-12-17

**Authors:** Albert Sotto, Frédéric Laurent, Sophie Schuldiner, Julien Vouillarmet, Stéphane Corvec, Pascale Bemer, David Boutoille, Catherine Dunyach-Rémy, Jean-Philippe Lavigne

**Affiliations:** 1 Virulence Bactérienne et Infections Chroniques, INSERM U1047, Université de Montpellier, Service de Maladies Infectieuses et Tropicales, CHU Nîmes, 30908 Nîmes, France; albert.sotto@chu-nimes.fr; 2 CIRI—Centre International de Recherche en Infectiologie, Inserm, U1111, Université Claude Bernard Lyon 1, CNRS, UMR5308, École Normale Supérieure de Lyon, Université Lyon, 69007 Lyon, France; frederic.laurent@univ-lyon1.fr; 3 VBIC, INSERM U1047, Université de Montpellier, Service des Maladies Métaboliques et Endocriniennes, CHU Nîmes, CEDEX 09, 30029 Nîmes, France; sophie.schuldiner@chu-nimes.fr; 4 Service d’Endocrinologie, Diabétologie et Nutrition, Hôpital Lyon-Sud, Hospices Civils de Lyon, 69002 Lyon, France; julien.vouillarmet@chu-lyon.fr; 5 Service de Bactériologie et des Contrôles Microbiologiques, CHU de Nantes, 44093 Nantes, France; stephane.corvec@chu-nantes.fr; 6 Department of Microbiology, University Hospital of Nantes, 44093 Nantes, France; pascale.bemer@chu-nantes.Fr; 7 Department of Infectious Diseases, CIC UIC 1413 INSERM, University Hospital, 44000 Nantes, France; david.boutoille@chu-nantes.fr; 8 Virulence Bactérienne et Infections Chroniques, INSERM U1047, Université de Montpellier, Service de Microbiologie et Hygiène Hospitalière, CHU Nîmes, 30908 Nîmes, France; catherine.remy@chu-nimes.fr

**Keywords:** Antibiofilmogram, antibiotics, biofilm, diabetic foot infections, *Staphylococcus aureus*, wound healing

## Abstract

Infected diabetic foot ulcers (DFUs) represent a serious threat to public health because of their frequency and the severity of their consequences. DFUs are frequently infected by bacteria in biofilms, obstructing antibiotic action. Antibiofilmogram was developed to assess the impact of antibiotics to inhibit biofilm formation. This pilot study aimed to determine the benefits of this technology in predicting antibiotic activity on the outcome of 28 patients with Grade 2 DFUs that were infected by a monomicrobial *Staphylococcus aureus*. Patients with diabetes were followed during the antibiotic treatment (day 14) and the follow-up period of the study (day 45). The contribution of Antibiofilmogram was compared between patients with non-concordant results (*n* = 13) between antibiogram and Antibiofilmogram versus concordant results (*n* = 15). The clinical improvement of wounds (80.0% vs. 38.5%, *p* = 0.0245) and the absence of exudates (0% vs. 33.3%, *p* = 0.0282) were observed in concordant vs. discordant groups. This pilot study provides promising results for the interest of Antibiofilmogram in the prescription of antibiotics to prevent biofilm formation in infected DFUs.

## 1. Introduction

Foot ulceration is one of the most frequently recognized complications in patients living with diabetes, as an ultimate result of a triopathy associating sensory, autonomic, and motor neuropathies, immunopathy, and lower limb arteriopathy [1]. Infection of these ulcers is a frequent (40–80%) and costly complication, increasing diabetes-related hospital admissions, mortality, and morbidity [1]. The management of this complication is a challenging problem, and wound-healing outcomes are often poor [2].

Diabetic foot ulcers (DFUs) are often infected with commensal and pathogenic microorganisms, especially containing *Staphylococcus* spp. [3,4]. For clinicians, the difficulties in distinguishing between infection and colonization of DFUs frequently leads to non-adapted antimicrobial treatment with overly broad-spectrum or excessively prolonged treatment [1]. This increases the risk of non-traumatic lower limb amputations [1] and the emergence of multidrug-resistant organisms [1,3,4,5,6]. Moreover, among these chronic wounds, 60–80% of microorganisms are organized in biofilm, increasing the difficulty of treating these lesions because sessile bacteria have a higher tolerance towards antibiotics, and bacterial biofilms play a crucial role in delayed wound healing [7,8,9]. It is also known that some antibiotics have an inductive effect around therapeutic doses on biofilm behavior, with consequences for the duration of remission and/or recurrence of the wound infections [10].

To date, clinicians have no available routine information or tools to investigate the role of antibiotics in wound healing, or to predict wound evolution. Recently, a diagnostic tool derived from the BioFilm ring test (BioFilm Control, St Beauzire, France) was elaborated to investigate the capacity to study the early biofilm formation of bacteria, and it has been used to assess the impact of antibiotics to inhibit the installation of this early biofilm formation [11]. Antibiofilmogram provides complementary information for the traditional antibiogram in order to decipher the efficiency of antibiotics against biofilm formation. Here, we conducted a pilot study to test the benefit of Antibiofilmogram use for the clinicians, providing information on the efficiency of antibiotics against biofilm formation regarding the risk of failure of an antibiotic regimen on the evolution of DFUs infected by *S. aureus.*

## 2. Materials and Methods

### 2.1. Study Design

This prospective, multicenter, observational pilot study was approved by the South Mediterranean III Ethics Committee (clinicaltrials.gov (accessed on 30 January 2020) #NCT02378493). From 16 December 2015 to 14 July 2019, we enrolled persons with diabetes who were admitted to three French diabetic foot clinics (Nîmes, Nantes, and Lyon) with a suspected new episode of diabetic foot infection (DFI) (Grade 2, according to the PEDIS (Perfusion Extent, Depth, Infection, Sensation) classification of the International Working Group of Diabetic Foot (IWGDF) consensus conference [1]), without antibiotic treatment in the past 14 days and with monomicrobial culture of *S. aureus*. The presence and severity of the infections was assessed by a trained diabetologist or infectious disease specialist. Demographic, comorbidities and clinical data were collected in this study. Arteriopathy was clinically assessed by the presence or absence of suggestive symptoms, such as intermittent claudication or leg pain at rest, and signs such as cold legs or feet, pale or bluish color of the skin, and foot pulses. In addition, according to local usual practices, a Doppler ultrasound examination, ankle-brachial pressure index (ABPI), and transcutaneous oxygen pressure (TcPO_2_) were implemented. Neuropathy was assessed by the presence or absence of paresthesia or cramps, as well as dry skin or hyperkeratosis of the foot, Charcot foot, and other foot deformities and protective sensation (using the 10 g Semmes–Weinstein monofilament testing, as recommended by the IWGDF). After wound debridement, samples of the bacterial cultures were obtained by scraping and swabbing at the wound base, or by tissue biopsies [1]. Antibiotics were prescribed for 14 days following the local protocol of each hospital and the IWGDF recommendations [1]. Each center has followed its own protocol to manage the wounds. All patients had general measures, including a prescription for offloading devices, dressings changed by nurses, the controlling of blood glucose, and an anti-tetanus vaccination if needed. Patients were followed-up on days 14 and 45. Wound evolution was assessed via surface area and depth and the presence of inflammatory signs and exudates. An outcome was considered ‘unfavorable’ in patients seeking an early review and for worsening/stagnating wounds or ‘favorable’ for completely or partially re-epithelialized wounds. The definition of healing was based on the criterion of reaching at least a 40% reduction of the initial ulcer area at the end of the study (day 45), as previously proposed by Edmonds et al. [12] and the French High Authority of Health [13].

### 2.2. Bacteriological Study

The isolates were identified using the Vitek^®^ MS system (bioMérieux, Marcy L’Etoile, France). Antimicrobial susceptibility testing was performed by a disk diffusion test (Bio-Rad, Marnes-la-Coquette, France) or broth microdilution procedures (UMIC) (Biocentric, Bandol, France), according to EUCAST recommendations (https://www.eucast.org/clinical_breakpoints (accessed on 10 September 2021)). Vancomycin and teicoplanin MICs were determined using the broth microdilution procedures (UMIC) (Biocentric, Bandol, France).

Multilocus sequence typing (MLST) was performed on *S. aureus* on inclusion, day 14, and day 45 [14]. Seven housekeeping genes (*arc*, *aroE*, *glpF*, *gmk*, *pta*, *tpi* and *yqi*) were sequenced to determine the allelic profile. The strains were assigned to an ST using the MLST database [15].

### 2.3. Antibiofilmogram

The Biofilm ring test was used to study antibiotic action on biofilm formation and to determine an Antibiofilmogram (BioFilm Control), as previously described [10]. Briefly, experiments were performed with the bacterial isolate using the brain heart infusion medium. The 96-well microtiter plates containing bacteria, magnetic beads and antibiotics (20 μL of antibiotic solutions) were incubated at 37 °C for 4 h before visual reading. At this time, the plates were placed onto a magnetic block, read after magnetic attraction (1 min), and analyzed using a microplate scanner with the BioFilm Control software (BFC Elements 3.0), which generated a biofilm formation index (BFI). Using a second algorithm (Algo CMIb), the biofilm minimal inhibitory concentration (bMIC) was assessed for 13 antibiotics (cloxacillin, ceftazidime, amoxicillin/clavulanic acid, teicoplanin, vancomycin, fosfomycin, ofloxacin, rifampicin, cotrimoxazole, gentamicin, clindamycin, erythromycin and fusidic acid). The bMICs were determined based on the BFIs using an algorithm developed and validated in-house. Four wells without antibiotics, filled with the bacterial suspension and magnetic beads, were used as the positive control (there was an absence of spots, due to beads immobilization in biofilm). Assays were performed in triplicate. The interpretations were performed by comparing the bMICs obtained with the EUCAST breakpoints (V 1.1 April 2020; www.eucast.org/clinical_breakpoints (accessed on 10 September 2021)). The oxacillin breakpoint was used as a proxy amoxicillin/clavulanic acid and ceftazidime breakpoint. The final result was communicated to the clinician at the end of the study and was a susceptibility classification of the particular strain (sensitive, intermediate, resistant) toward the selected antibiotics.

### 2.4. Statistical Analysis

The primary outcome was the role of antibiogram/Antibiofilmogram concordance (in terms of *S. aureus* strains and prescribed antibiotics) on the presence/absence of *S. aureus* strains on day 14 (at the end of antibiotic treatment). The secondary outcome was the role of antibiogram/Antibiofilmogram concordance in wound improvement and healing. Patients were classified to the concordant group if all the antibiotics prescribed were active against *S. aureus* and efficient against the biofilm formation, or the discordant group if one or two antibiotics were inactive against the *S. aureus* strain or inefficient against biofilm formation.

This study was exploratory; however, the inclusion of 32 patients would allow us to demonstrate a relative risk (RR: the probability of absence of *S. aureus* at the end of antibiotic therapy in case of concordance/the probability of absence of *S. aureus* at the end of antibiotic therapy in case of discordance) equal to 2 for a concordance rate between 43% (e.g., teicoplanin), and 71% (e.g., vancomycin); and an RR of 3 for a concordance rate of 88% (e.g., erythromycin/fusidic acid), with a power of 80% and a bilateral alpha risk of 5%—taking into account the consecutive inclusion of patients and considering a 15% rate of patients with non-exploitable data. These data, used for sample size calculation, came from preliminary data using the BFC software and were obtained with an antibiotic panel chosen by the investigators from Nîmes University Hospital. The concordance rate by antibiotic was very variable and depended on the antibiotics tested: 5% to 95% (unpublished laboratory data), but mostly higher than 40%. We expected a relatively large concordance effect on the absence of *S. aureus* at the end of antibiotic therapy, but this is not yet quantified.

The normality of the quantitative variables’ distribution was determined using the Shapiro–Wilk normality test with a threshold of 0.01 and coefficients of kurtosis and skewness. Statistical results were to be presented as the means ± standard deviations (SD) for quantitative variables following a Gaussian distribution, and means and 95% back-transformed confidence intervals for Gaussian variables after transformation. Medians and interquartile (IQ) ranges were used for the other variables. For the qualitative variables, the numbers and the associated percentages were to be presented. A univariate analysis was determined concerning patient characteristics at inclusion and the rate of absence of *S. aureus* at D14 between groups. Qualitative variable comparisons were carried out using a chi-square test or Fisher’s exact test. Quantitative variable analyses between the two groups were performed using a Kruskal–Wallis test. DFU healing at the end of the study was compared between the two groups via the chi-square test or Fisher’s exact test.

The potential role of Antibiofilmogram and the pre-defined cofactors in predicting wound evolution was studied between groups. The scores established from this matrixial analysis were compared between two groups and certified by Soladis (Lyon, France). A comparison of wound evolution on day 14 and day 45 between groups was performed using a chi-square or Fisher’s exact test for qualitative variables, and a Student’s test or Wilcoxon rank sum test was used for quantitative variables. The individual trajectories of clinical course wound area and depth during the study were represented graphically. The statistical analysis was to be conducted under the SAS (SAS institute, Cary, NC, USA) version 9, or R 2.9.2 (R development Core Team 2009, R foundation for Statistical Computing, Vienna, Austria). Differences were considered statistically significant when the degree of significance (*p*-value) of the test was ≤0.05.

## 3. Results

### 3.1. Studied Population

**Figure 1:**
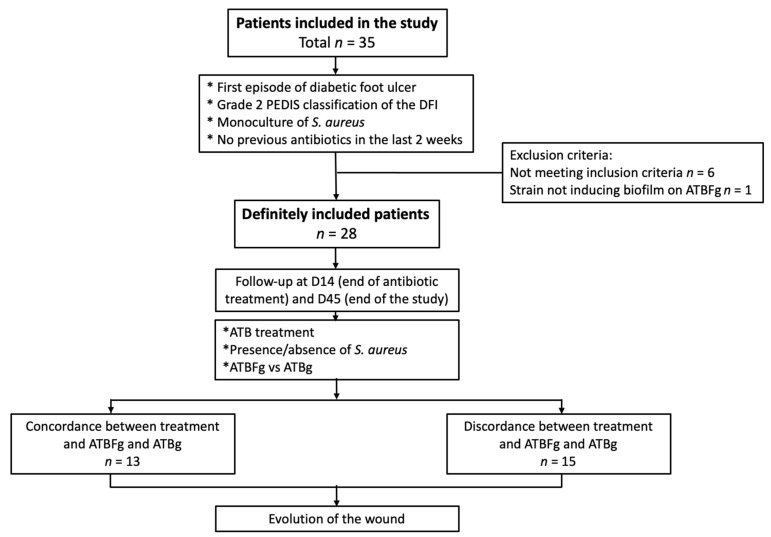
Flowchart of the study. ATBFg, Antibiofilmogram; ATBg, antibiogram.

Thirty-five patients were screened, with seven excluded due to the problem of bacterial identification at inclusion (*n* = 2), bacterial conservation (*n* = 2), antibiotic therapy in the last 2 weeks (*n* = 2) and non-formation of biofilm with Antibiofilmogram (*n* = 1) (Figure 1). Finally, 28 patients were definitively included: *n* = 18 at Nîmes, *n* = 7 at Lyon and *n* = 3 at Nantes.

Most of the included patients were male (22, 78.6%) with a mean age of 61.2 years (±11.92) and type 2 diabetes (26, 92.9%) (Table 1). The median Charlson score was 3 (±2). The median wound surface area was 119 mm^2^ (±197.65), and the median depth was 3 mm (±9), with exudates in 6 wounds (24%).

Seventeen patients (60.7%) received bitherapy (Table 1). The main antibiotics administered were β-lactams (*n* = 19, 67.9%), notably amoxicillin/clavulanic acid (*n* = 15, 53.6%), followed by clindamycin (*n* = 9, 32.1%), ofloxacin (*n* = 6, 21.4%), and rifampicin and cotrimoxazole (*n* = 5, 17.9%).

The bacteriological analysis identified 28 *S. aureus* at inclusion. Seven (25%) patients had *S. aureus* infection on day 14 and 9 (32.1%) on day 45. A total of 44 isolates were analyzed by Antibiofilmogram.

Thirteen patients showed discordant results and 15 concordant between the antibiogram and Antibiofilmogram (Table 2). Groups were demographically similar; however, monotherapy was more common and of a shorter duration in the concordant group (60.0% monotherapy vs. 15.4%, *p* = 0.0238; and 13 ± 5 days vs. 14 ± 3.5, *p* = 0.0393, respectively) (Table 1). Cotrimoxazole, ofloxacin and rifampicin were exclusively used in the discordant group (0% vs. 38.5%; 0 vs. 46.2%; 0 vs. 38.5%). Using Antibiofilmogram, clindamycin (9 strains/9) and rifampicin (3/3) were always efficient against biofilm formation, whereas ofloxacin (6/6), cotrimoxazole (5/5), and vancomycin (1/1) never were (Table 2).

### 3.2. Presence of S. aureus during the Follow-Up of the Patients

No significant differences were observed for *S. aureus* presence at the follow-up between the discordant (*n* = 3, 23.1% at day 14 and *n* = 4, 30.8% at day 45) and concordant groups (*n* = 4, 26.7% at day 14 and *n* = 5, 33.3% at day 45) (*p* = 0.574) (Table S1). The seven *S. aureus* isolated at inclusion and day 14 belonged to the same ST, suggesting that the strains were identical. On day 45, seven *S. aureus* always belonged to the same ST with two (C03P008 and C04P004) present in the three samples (inclusion, day 14 and day 45). In two cases (C04P005 and C06P003) a new ST was detected.

### 3.3. Antibiofilmogram and Evolution of the DFU

On day 14, fewer wounds were exudative in the concordant group (0% vs. 30.8%, *p* = 0.0282). Moreover, these patients showed clinical improvement (80.0% vs. 38.5%, *p* = 0.0245) and reduced wound depth (2 mm ±1.25 vs. 3 ±14.0), but these results were not significant (*p* = 0.0516). A non-significant greater diversification of species was isolated in the concordant group (1.79 vs. 1.58) (Table 3).

**Figure 2:**
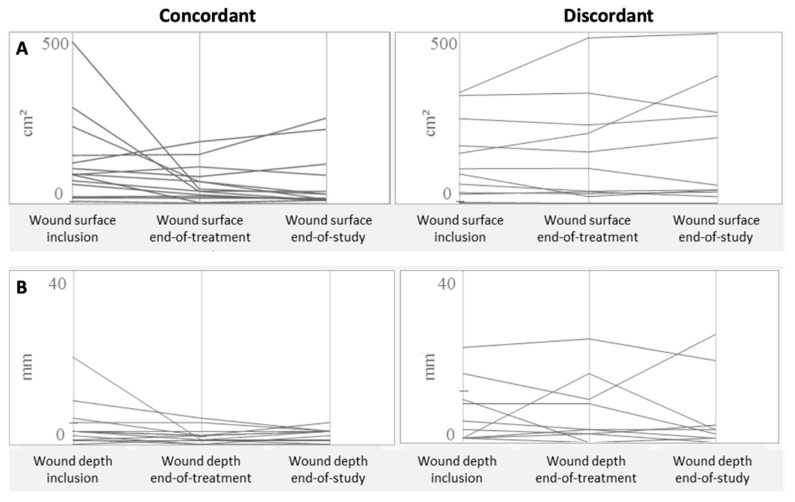
Individual evolution of the wound surface area (**A**) and wound depth (**B**) measurements at inclusion, at the end of treatment (day 14) and at the end of the follow-up (day 45) of patients with DFU infected by *S. aureus* and belonging to discordant and concordant groups based on the results of the Antibiofilmogram.

The representation of each individual evolution of the wounds showed that most patients in the concordant group had improved DFUs on day 14, in contrast to the discordant group (Figure 2). A clear amelioration of the wound surface and depth was noted in the concordant group (*n* = 12 with full or partial wound healing and *n* = 3 with stabilization/worsening) on day 45. The evolution in the discordant group was diverse: improvement (*n* = 8) and stabilization or aggravation (*n* = 5).

**Figure 3:**
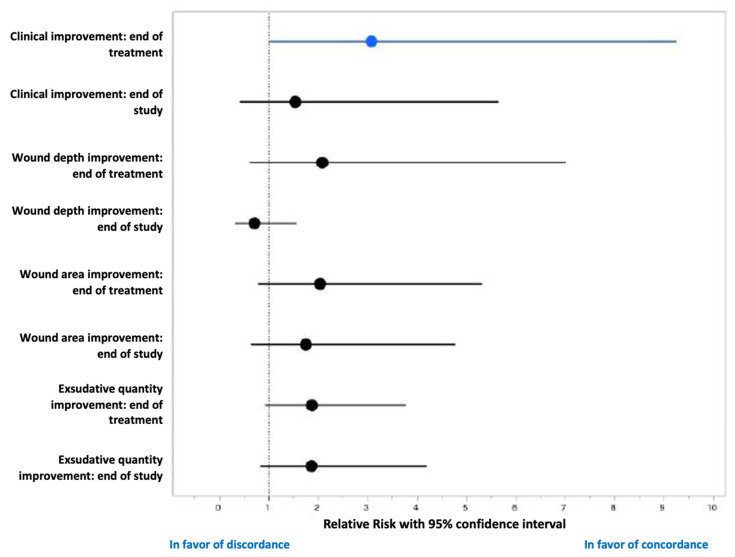
Forest plot (relative risk and 95% confidence interval) presenting the effect of the antibiogram/Antibiofilmogram concordance on the wound evolution.

Finally, at the end of the antibiotic treatment (day 14), 17 patients had favorable wound evolution (with 12 patients experiencing healing) and 11 unfavorable. An antibiogram/Antibiofilmogram concordance was noted in the patients with a favorable evolution, with a relative risk of 3.1 (95% CI:1–9.2) (Figure 3).

## 4. Discussion

Many factors influence the healing of DFUs. Among them, the polymicrobial biofilm represents one of the causes of delayed healing [7]. This non-healing of DFU appears to arise from a bacterial biofilm at the wound bed [7,16,17] and the organization of microorganisms in functionally equivalent pathogroups [7,18]. These sessile bacteria are difficult to treat, and few antibiotics are effective [19,20]. Standard antibiograms have limited ability to determine antibiotic effectiveness at the site of infection and on sessile bacteria. Recently, the Antibiofilmogram, based on the use of the BioFilm ring test [11,21], was adapted to evaluate the ability of antibiotics to inhibit biofilm growth [11]. Our first pilot multicenter re-study of the Antibiofilmogram contribution to guide clinicians in the treatment of DFU infected with *S. aureus* demonstrated promise for the clinical evolution of these wounds. The concordance between an antibiogram and Antibiofilmogram (meaning that the antibiotic would be effective against both planktonic and biofilm-form) was associated with the clinical improvement of the wound, fewer exudates at the end of antibiotic treatment (day 14), and a decreased wound area at the end of the follow-up (day 45).

Among the concordant group (concordance between antibiogram and Antibiofilmogram), a large majority of patients had a clinical improvement of their wound (*n* = 12/15, 80%) (Table 3). When we focused our attention on the three remaining patients, we noted that two had a worsening evolution of their wounds on day 14 (C01P004) and on day 45 (C04P004) (Figure 2), due to the presence of *P. aeruginosa* and *P. aeruginosa* + *K. oxytoca*, respectively. As these two patients received clindamycin alone (always efficient on biofilm installation) and amoxicillin/clavulanic acid alone (efficient on the biofilm installation of C04P004), we concluded that the two regimens were not adapted to treat the Gram-negative bacilli, even if recently Orazi et al. showed that *P. aeruginosa*-secreted products could increase the antibiotic activity to kill *S. aureus* in biofilm [22]. In the third case (C03P004), the administration of clindamycin alone seemed to be adapted, while *S. aureus* was not detected on day 14 and day 45, whereas the wound was stabilized but not improved. Only *Corynebacterium striatum*, a commensal bacterium of the skin, was detected in the follow-up of the patient. We could not exclude that other parameters, such as offloading, were not correctly applied, and that they influenced the wound evolution. A comparison between the two groups also showed a more important diversity in the number of bacterial species isolated from DFU in the concordant group, compared to the discordant group (1.79 vs. 1.58, respectively) during the follow-up of the wounds. One hypothesis should be that debridement is associated with a remodeling of cutaneous microbiota and that numerous species can colonize the wounds, explaining this greater bacterial diversification that could protect the ulcer to pathogenic bacteria and prevent infections.

Interestingly, only 7 patients (25%) presented persistent DFU colonization on day 14 and day 45 by a related ST strain. This constatation is in accordance with a recent observation performed in our hospital, where 25% of our panel (*n* = 48) harbored a related *S. aureus* isolate during a period of four weeks, with a median persistence of 12 weeks, and only one patient (2.1% of our panel) presented a successive *S. aureus* belonging to a same clonal lineage over time for an extended period exceeding 30 weeks [23]. This suggested that long-term persistence of *S. aureus* in DFI has a weaker implantation rate compared to other chronic conditions [24]. The debridement and antibiotic therapy could explain this low rate, yet the debridement appears to be sometimes insufficient, and many factors influence healing in persons with diabetes (e.g., offloading, antibiotic uptake, and glycemic balance). The MLST results also confirmed the important diversity of the *S. aureus* clones, as previously noted [25,26,27]. No clone was associated with the worsening evolution or an *S. aureus* persistence in the wound.

The main study limitation is the small size of our population (*n* = 28). The inclusion criteria were restrictive with only Grade 2—whereas, our specialized clinics followed mainly Grade 3 and 4 DFI and a monoinfection to *S. aureus*—and these wounds were preferentially polymicrobial [3,6,7]. Some antibiotics were more effective against biofilm infections and usable alone (β-lactams or related macrolides), or in combination (with rifampicin), but always with the need of the Antibiofilmogram. Larger prospective studies should confirm the value of Antibiofilmogram.

In conclusion, although not all of our outcomes showed significant differences—due to the small-sized cohort—our findings may suggest that an antibiotic strategy, which also incorporates information regarding antibiotic action against biofilms, may be a promising approach to improving wound healing outcomes for patients with DFUs.

## Figures and Tables

**Table 1:** Demographic and clinical characteristics of the study population at inclusion.

Characteristics	Concordant Group (*n* = 15)	Disconcordant Group (*n* = 13)	Total (*n* = 28)	*p*-Value Concordant vs. Disconcordant
Age (years, SD ^a^)	60.1 (±13.1)	62.4 (±10.9)	61.2 (±11.9)	0.6273
Male/Female (*n*,%)	11 (73.3)/4 (26.7)	11 (84.6)/2(15.4)	22 (78.6)/6 (21.4)	0.4865
BMI ^b^ (kg/m^2^, SD)	29.93 (±5.18)	33.67 (±7.94)	31.66 (±6.75)	0.1462
Comorbidities				
Charlson index (median, IQ ^c^)	2 (3)	4 (1.5)	3 (2)	0.4591
McCabe Score	1	1	1	>0.99
Arteriopathy (*n*,%)	14 (93.3)	12 (92.3)	26 (92.3)	>0.99
Neuropathy (*n*,%)	13 (86.7)	12 (92.3)	25 (89.3)	>0.99
Diabetes duration median (years, IQ)	15 (±10)	16 (±10)	15.5 (±10.7)	0.473
HbA1c mean (%, SD)	8.60 (±2.18)	7.93 (±1.4)	8.29 (±1.86)	0.3536
Type 1/Type 2 diabetes mellitus (*n*,%/*n*,%)	1 (6.7)/14 (93.3)	1 (7.7)/12 (92.3)	2 (7.1)/26 (92.9)	0.9201
Characteristics of the wounds				
Initial wound depth median (mm, IQ)	3 (±4.25)	7 (±14)	3 (±9)	0.2621
Initial wound surface area median (mm^2^, IQ)	117.8 (±168.8)	120.1 (±265.75)	119 (±197.65)	0.9632
Exsudative wound (*n*,%)	2 (13.3)	4 (30.8)	6 (24)	0.3720
Monotherapy/Bitherapy (*n*,%)	9 (60.0)/6 (40.0)	2 (15.4)/11 (84.6)	11 (39.3)/17 (60.7)	**0.0238**
Treatment duration (day, SD)	13 ± 5	14 ± 3.5	13 ± 3.8	**0.0393**
β-lactams (*n*,%)	10 (66.7)	9 (69.2)	19 (67.9)	0.7051
Macrolides and related (*n*,%)	7 (46.7)	3 (23.1)	10 (35.7)	0.1145
Cotrimoxazole (*n*,%)	0 (0)	5 (38.5)	5 (17.9)	**0.0131**
Glycopeptides (*n*,%)	0 (0)	2 (15.4)	2 (7.1)	0.2063
Fluoroquinolones (*n*,%)	0 (0)	6 (46.2)	6 (21.4)	**0.0046**
Rifampicin (*n*,%)	0 (0)	5 (38.5)	5 (17.9)	**0.0131**

^a^ SD, standard deviation; ^b^ BMI, body mass index; ^c^ IQ, interquartile; *p*-value was calculated using the Student test for demographic data, the Wilcoxon test for the Charlson score, the diabetes duration, the characteristics of the wounds and the treatment duration, and the Fisher exact test for the other variables. In bold, significant results (*p* < 0.05).

**Table 2:** Results of antibiogram and Antibiofilmogram of *S. aureus* strains isolated from DFI against the final antibiotics prescribed.

Classification Group	Patients	Antibiotics Prescription ^a^	Result of Antibiogram ^b^	Results of Antibiofilmogram
Concordant	C01P004	CLN	S	S
	C01P009	CLN	S	S
	C01P012	AMC	S	S
	C03P001	AMC	S	S
	C03P003	CLN	S	S
	C03P004	CLN	S	S
	C03P006	CLN	S	S
	C03P008	AMC and CLN	R/S	R/S
	C03P009	CLN	S	S
	C03P010	AMC and CLN	R/S	R/S
	C04P002	AMC	R	R
	C04P003	AMC	S	S
	C04P004	AMC	S	S
	C04P005	AMC	S	S
	C06P001	AMC	S	S
Discordant	C01P001	OFX + RIF	S/S	R/S
	C01P002	AMC + OFX	S/S	S/R
	C01P005	AMC + OFX	S/S	S/R
	C01P008	AMC + SXT	S/S	R/R
	C01P010	OFX + CLN	S/S	R/S
	C01P011	SXT + OFX	S/S	R/R
	C01P013	SXT + RIF	S/S	R/S
	C03P002	OFX + SXT	S/S	R/R
	C03P007	CLN + VAN	S/S	S/R
	C04P001	SXT	S	R
	C04P007	AMC	S	R
	C04P008	AMC + RIF	S/S	R/S
	C06P003	AMC	S	R

^a^ AMC, amoxicillin/clavulanic acid; CLN, clindamycin; OFX, ofloxacin; RIF, rifampicin; SXT, cotrimoxazole; VAN, vancomycin; ^b^ S, susceptible; R, resistant.

**Table 3:** Evolution of the DFU infected by *S. aureus* at the end of treatment (day 14) and at the end of the follow-up (day 45).

Characteristics	Concordant Group (*n* = 15)	Discordant Group (*n* = 13)	Total (*n* = 28)	*p*-Value Concordant vs. Discordant
End of treatment (Day 14)				
Wound depth median (mm, IQ ^a^)	2 ± 1.25	3 ± 14	2 ± 4.5	0.0516
Wound surface area (mm^2^, IQ)	43.55 ± 72.12	75.8 ± 220.15	43.55 ± 135.8	0.4556
Exsudative wound (*n*, %)	0 (0)	4 (30.8)	4 (14.3)	**0.0282**
Inflammatory signs (*n*, %)	8 (53.3)	11 (84.6)	19 (67.9)	0.0823
Number of species (mean, SD ^b^)	1.79 ± 1.05	1.58 ± 0.51	1.69 ± 0.84	0.55
Gram-Negative Bacilli (*n*, %)	5 (33.3)	6 (46.2)	11 (39.3)	0.6922
Gram-Positive Cocci (*n*, %)	8 (53.3)	7 (53.8)	15 (53.6)	>0.999
Anaerobes (*n*, %)	0 (0)	0 (0)	0 (0)	>0.999
Clinical improvement (*n*, %)	12 (80.0)	5 (38.5)	17 (60.7)	**0.0245**
Wound healing (*n*, %)	8 (53.3)	4 (30.8)	12 (42.9)	0.2219
End of follow-up (Day 45)				
Wound depth median (mm, IQ)	3 ± 2	3 ± 18	3 ± 2	0.5482
Wound surface area (mm^2^, IQ)	22 ± 70.2	42.4 ± 185.3	29.85 ± 157.62	0.0595
Exsudative wound (*n*, %)	2 (13.3)	3 (20.0)	5 (17.9)	0.3217
Inflammatory signs (*n*, %)	7 (46.7)	9 (69.2)	16 (57.1)	0.4404
Number of species (mean, SD)	1.87 ± 1.13	1.69 ± 0.75	1.79 ± 0.96	0.6395
Gram-Negative Bacilli (*n*, %)	5 (33.3)	3 (20.0)	8 (28.6)	0.686
Gram-Positive Cocci (*n*, %)	10 (66.7)	7 (53.8)	17 (60.7)	0.7
Anaerobes (*n*, %)	0 (0)	3 (20.0)	3 (10.7)	0.0873
Clinical improvement (*n*, %)	12 (80)	8 (61.5)	21 (75.0)	0.5295
Wound healing (*n*, %)	11 (73.3)	8 (61.5)	19 (67.9)	0.4953

^a^ IQ, interquartile range; ^b^ SD, standard deviation; *p*-value was calculated using the Wilcoxon test for wound characteristics and the Fisher exact test for the other variables. In bold, significant results (*p* < 0.05).

## Supplementary Materials

The following are available online at https://www.mdpi.com/article/10.3390/jcm10245928/s1, Table S1: Comparison of MLST results of the *S. aureus* strains isolated from DFI at inclusion, at the end of the treatment, and at the end of the study.
