# Supplementary material for: Evaluation of the Use of Antibiofilmogram Technology in the Clinical Evolution of Foot Ulcers Infected by Staphylococcus aureus in Persons Living with Diabetes: A Pilot Study
Source: J Clin Med. 2021 Dec 17;10(24):5928. doi: 10.3390/jcm10245928 (PMC8705769; doi:10.3390/jcm10245928)
Supplement: Supplementary file 1 [file jcm-10-05928-s001.zip › jcm-1480572-supplementary.pdf]

Table S1. Comparison of MLST results of the *S. aureus* strains isolated from DFI at inclusion, at the end of the treatment and at the end of the study.

**Patient**	***S. aureus* ST:**		
	**At Inclusion**	**At the End of Treatment D15**	**At the End of Study D45**
C01P001 (Case)	ST45	ST45	-
C01P002 (Case)	ST97		ST97
C01P010 (Case)	ST30		ST30
C01P011 (Case)	ST97	ST97	
C01P013 (Case)	ST59		ST59
C03P002 (Case)	ST2199	ST2199	-
C03P008 (Control)	ST30	ST30	ST30
C03P010 (Control)	ST5		ST5
C04P002 (Control)	ST149		ST149
C04P004 (Control)	ST30	ST30	ST30
C04P005 (Control)	ST45	ST45	ST398
C06P001 (Control)	ST15	ST15	
C06P003 (Case)	ST398		ST2434
